# Physicochemical Factors That Influence the Biocompatibility of Cationic Liposomes and Their Ability to Deliver DNA to the Nuclei of Ovarian Cancer SK-OV-3 Cells

**DOI:** 10.3390/ma14020416

**Published:** 2021-01-16

**Authors:** Mengwei Sun, Yuhao Yuan, Fake Lu, Anthony J. Di Pasqua

**Affiliations:** 1 Department of Pharmaceutical Sciences, School of Pharmacy and Pharmaceutical Sciences, Binghamton University, 96 Corliss Ave., Johnson City, NY 13790, USA; msun22@binghamton.edu; 2 Department of Biomedical Engineering, The Thomas J. Watson College of Engineering and Applied Science, Binghamton University, 4400 Vestal Pkwy. E., Binghamton, NY 13902, USA; yyuan22@binghamton.edu (Y.Y.); fakelu@binghamton.edu (F.L.)

**Keywords:** liposome, DNA delivery, ovarian cancer

## Abstract

Cationic liposomes composed of 3-[N-(N’,N’-dimethylaminoethane)-carbamoyl] cholesterol (DC-chol) and dioleoylphosphatidylethanolamine (DOPE) have previously been shown to have applications in gene delivery. Our study aims to explore the effects of inclusion of polyethylene glycol (PEG) and using different molar ratios of DC-chol/DOPE on size, zeta potential, cytotoxicity and DNA delivery of DC-chol/DOPE liposomes. Our results show that PEGylation reduces the cytotoxicity of DC-chol/DOPE liposomes, and, furthermore, PEGylated liposome-DNA lipoplexes are smaller in size and more uniform in size distribution than those that are not PEGylated. Additionally, toxicity against ovarian cancer SKOV-3 cells decreases with the amount of cationic DC-chol present in the formulation; however, decreased delivery of DNA to cellular nuclei is also observed. Transfection with the PEGylated liposomes was successfully demonstrated using plasmid DNA with a known functional outcome. These results offer further insight into physicochemical properties important for cationic liposomes as vehicles for DNA delivery and demonstrate the potential of PEGylated DC-chol/DOPE liposomes as systemic delivery carriers for DNA-mediated ovarian cancer therapy.

## 1. Introduction

The progress of gene therapy depends on productive and reliable systems for the delivery of exogenous DNA or RNA into target cells. Lipid-based nonviral systems for gene transfer have been widely investigated over the past few decades. Composed of relatively biocompatible and biodegradable materials, liposomes have aqueous cores and at least one bilayer of natural and/or synthetic lipids, making them capable of enhancing the efficacy and improving the biocompatibility of the encapsulated gene/drugs [1,2,3]. Efficient gene delivery capacity has been demonstrated by cationic liposomes and therefore they have been extensively utilized as gene carriers [4,5,6,7,8,9]. The electrostatic interactions between positively charged liposomes and negatively charged cell membranes usually support liposome internalization into cells and consequently facilitate DNA delivery to the nucleus [10]. However, the in vivo application of liposomal delivery systems is often hindered by their instability in serum and limited circulation times, since the cationic surfaces of liposomes also tend to interact electrostatically with serum proteins and components related to the immune system. Moreover, the DNA complexed on the surface of liposomes might suffer from rapid degradation via biological defense systems such as endogenous DNase activity. Surface modifications with polyethylene glycol (PEG), a hydrophilic polymer, have been recently proved to improve liposomal gene delivery. PEGylated liposome-DNA complexes are able to aid in increased transfection efficiencies in the presence of serum, compared to nonPEGylated ones [11]. Furthermore, the PEGylated complexes can build a steric barrier to enhance stability and prolong circulation time and may help to diminish aggregation issues of the liposomes by mutually repulsive interactions between the PEG molecules [12,13,14].

When using a lipoplex, an effective gene transfer process faces several barriers, including the internalization of the lipoplex and intracellular trafficking, such as endosomal escape and nuclear entry [15,16]. The addition of cholesterol in a lipoplex formulation has been confirmed to enhance transfection both in cell culture and in vivo, due to the formation of cholesterol nanodomains on the lipoplex membrane, which can affect lipoplex internalization and intracellular trafficking [17,18,19]. In addition, cholesterol can decrease the interaction between serum proteins and lipoplexes, which is attributed to the impact of cholesterol on the fluidity of cellular membranes or on the stability of lipoplexes in serum-containing media [20,21]. Many cholesterol derivatives have been synthesized to improve gene delivery and expression. As one of the most widely used derivatives, 3-[N-(N’,N’-dimethylaminoethane)-carbamoyl] cholesterol (DC-chol) has been reported to effectively transfect DNA in multiple studies [22,23,24].

Helper lipids (usually neutral) are frequently formulated with cationic lipids together to aid in the formation or structure of the liposome–DNA complex. Dioleoylphosphatidylethanolamine (DOPE), a neutral lipid, is often used in conjunction with cationic lipids, due to its hexagonal conformation which allows for efficient escape of complexed DNA from endosomal vesicles via destabilization of the vesicle membrane [25,26].

DC-chol/DOPE liposomes have been extensively investigated as gene transfection carriers; however, little insight is available regarding the effect of PEGylation on these delivery vehicles, and the optimal ratio of cationic/helper lipid for biocompatibility and transfection efficiency. In this study, we prepared and characterized PEGylated and un-PEGylated DC-chol/DOPE liposomes at five different DC-chol to DOPE molar ratios (3:1, 2:1, 1:1, 1:2, and 1:3). The toxicities of these formulations were then investigated against ovarian cancer SKOV-3 cells. Three relatively noninvasive formulations were complexed with fluorescent labeled oligonucleotides to examine their abilities to deliver DNA into the cell and the cell nuclei by Stimulated Raman Scattering (SRS) microscopy and a nuclear/cytoplasmic separation study. Finally, these three formulations were selected to deliver GFP-expressing plasmid (pDNA) to SK-OV-3 cells, to investigate their transfection efficiencies, as well as the influence of serum on their transfection ability. pDNA was used as it has a known functional outcome. Better understanding the influence of PEGylation and lipid molar ratios on cationic liposomes is expected to improve the design of liposomes for efficient and safe DNA delivery.

## 2. Materials and Methods

*Materials:* 3-[N-(N’,N’-dimethylaminoethane)-carbamoyl] cholesterol (DC-chol), 1,2-dioleoyl-sn-glycero-3-phosphoethanolamine (DOPE) and 1,2-distearoyl-sn-glycero-3-phosphoethanolamine-N-[amino(polyethyleneglycol)-2000] (DSPE-PEG-2000) were purchased from Avanti Polar Lipids, Inc. (Alabaster, AL, USA). Chloroform, McCoy’s 5a modified growth media and fetal bovine serum were purchased from Millipore Sigma (St. Louis, MO, USA). The single-stranded fluorescent labeled oligonucleotide clamp 5′–[6-FAM] CTCCTCCCATTTTTATAAG–3′ was obtained from Eurofins MWG Operon (Huntsville, AL, USA). Both this and the SK-OV-3 cells (purchased from the American Type Culture Collection (ATCC; Manassas, VA, USA)) were kindly gifted to us by Dr. Tracy Brooks (Binghamton University). The Aqueous One Solution Cell Proliferation Assay (MTS) was obtained through Promega (Madison, WI, USA). The 5× DNA loading buffers were purchased from Thomas Scientific (Swedesboro, NJ, USA). Hypotonic buffer, NP-40, and microcover glasses were purchased from VWR (Radnor, PA, USA). GFP-expressing plasmid DNA was purchased from Altogen Biosystems (Las Vegas, NV, USA). Lipofectamine^TM^ LTX Reagent was obtained from Invitrogen (Carlsbag, CA, USA).

*Preparation of liposomes and liposome-DNA complexes (lipoplexes):* PEGylated or nonPEGylated DC-chol/DOPE liposomes were prepared by the thin-film evaporation method. Briefly, DC-chol/DOPE at molar ratios of 3:1, 2:1, 1:1, 1:2 and 1:3, with and without 5% DSPE-PEG-2000, were dissolved in 2 mL chloroform in a round bottom flask to a total amount of 20 µmol, and the solvent was then evaporated under a vacuum by rotary evaporation for 30 min at 40 °C in order to form a thin lipid film. Then, 1 mL of 10 mM phosphate-buffered saline (pH = 7.4) was added to the film which was subjected to 30 s sonicating (Branson Ultrasonics Corporation, Danbury, CT, USA) and 60 s vortexing cycles at 40 °C until the film was completely hydrated and the solution was homogeneous. The multilamellar liposome solution was extruded through a 200 nm and then a 100 nm polycarbonate membrane 15 times at 40 °C using a manual extruder (Avanti Polar Lipid, Inc., Alabaster, AL, USA). The lipid content in the samples of extruded liposomes was not evaluated in the present study. It is expected that the lipid content of extruded samples would decrease due to extrusion through the membranes of small pore sizes [27,28]. The liposome sample was diluted with an equal volume of suspended DNA (5′–[6-FAM] CTCCTCCCATTTTTATAAG–3′) at a concentration of 300 µg/mL. DNA was added dropwise to the liposome solution and the liposome–DNA complex (lipoplex) was incubated for 30 min at room temperature under constant gentle mixing. The lipoplexes prepared had final lipid concentrations of 10 µM and final DNA concentrations of 150 µg/mL. Lipoplexes containing 0.5 μg pDNA, which were used in the transfection study, were prepared similarly.

*Characterization of blank liposomes and lipoplexes:* A Zetasizer Nano (Malvern Instruments, Worcestershire, UK) was used to investigate the particle size distribution and zeta potential of the blank liposomes and lipoplexes prepared using the dynamic light scattering technique. The DNA binding affinity of the lipoplexes was characterized by a gel retardation assay. Naked FAM-labeled DNA was included as a positive control. A 20 µL aliquot of each sample was mixed with 5 µL of 5 × DNA loading buffer and added into individual wells on a 1% (*w/v*) agarose gel, and electrophoresis was performed in Tris/Acetate/EDTA (TAE) buffer at 100 V for 30 min. The gel was then imaged with an Azure Imaging System (Azure Biosystems, Dublin, CA, USA) via EPI Blue excitation to image fluorescence signal in the samples.

*Cytotoxicity of blank liposomes:* All cell studies were carried out in a humidified 37 °C, 5% CO_2_ (standard conditions) atmosphere incubator. Human ovarian cancer SK-OV-3 cells were cultured with McCoy’s 5a modified growth media supplemented with 10% fetal bovine serum (FBS) and 1× penicillin-streptomycin. Eleven groups (*n* = 4) including ten treatment groups, PEGylated or nonPEGylated DC-chol/DOPE at molar ratios of 3:1, 2:1, 1:1, 1:2 and 1:3, and a Phosphate Buffer Saline (PBS) control group, were tested in the cell line. The cells were seeded at 5 × 10^3^ cells/well (100 µL/well) in 96-well plates and allowed to grow for 24 h. After this, the medium was removed and replaced with 100 µL of medium containing the blank liposomes samples. Following a 72 h incubation period, the old media were removed and replaced with 100 µL fresh media containing 20 µL of 3-(4,5-dimethylthiazol-2-yl)-5-(3-carboxymethoxyphenyl)-2-(4-sulfophenyl)-2H-tetrazolium (MTS) solution. The UV–Vis absorbance was read at 490 nm after 2 h of incubation. The percentage survival of cells treated was calculated using the following equation:

(1) $$\%\;\mathrm{Survival}=\;\frac{A_{t}-A_{m}}{A_{c}-A_{m}}\;\times 100\%$$

where *A_t_* is the absorbance of cells in treatment groups, *A_m_* is the absorbance of the medium alone and *A_c_* is the absorbance of cells in the PBS control group.

*Release of DNA from the lipoplex:* The in vitro DNA release profiles of PEGylated 1:1, 1:2 and 1:3 DC-chol/DOPE lipoplexes were tested and compared to free DNA control. Briefly, 1 mL of lipoplex or DNA control with the same DNA concentration was placed inside a tightly sealed 12 kDa MWCO dialysis bag (Sigma-Aldrich, St. Louis, MO, USA) and immersed in 10 mL of PBS (pH 7.4) in a closed glass vial. The temperature of the release system was maintained at 37 ± 0.5 °C with constant stirring at 100 rpm. At predetermined time points, aliquots (100 μL) were taken in triplicate from the release media and replaced with PBS (pH = 7.4). The amount of DNA released into the release media was determined via the fluorescence label of DNA, FAM, which has excitation and emission wavelengths of 495 and 520 nm, respectively. SpectraMax i3 (Molecular Devices, San Jose, CA, USA) was used for fluorescence detection. Cumulative release percentage of DNA over time was calculated using the equation:

(2) $$\begin{array}{l} \mathrm{Cumulative}\;\mathrm{Release}\;\left(\%\right)\\ \;=\;\frac{Cumulative\;amount\;of\;DNA\;released}{Total\;amount\;of\;DNA\;in\;the\;liposomes}\\ \;\;\times 100\% \end{array}$$

*The integrated stimulated Raman scattering (SRS) and two-photon excitation fluorescence (TPEF) microscopy system:* A lab-built SRS/TPEF microscope was used for cell imaging and DNA tracking using a dual-beam near-infrared femtosecond laser source (InSight X3, Spectra-Physics, Santa Clara, CA, USA). Label-free lipid imaging of the cells was performed at the Raman shift 2854 cm^−1^ by tuning the laser wavelength to 805 nm, which is attributed to the CH_2_ chemical bond vibration in lipids (pseudocolor green) [29]. Label-free protein imaging of the cells was performed at the Raman shift 2930 cm^−1^ by tuning the pump beam wavelength to 800 nm, which is attributed to the CH_3_ chemical bonds vibration in proteins (pseudocolor blue). At the same time, with the same 800 nm excitation, the backward two-photon fluorescence imaging of FAM-labeled DNA (pseudocolor red) was acquired using a photomultiplier tube (R10699, Hamamatsu Photonics, Japan) through a fluorescence emission filter (FF03-525/50-25, Semrock, Rochester, NY, USA). The SRS/TPEF imaging system was controlled by the ScanImage software (Vidrio Technologies, Ashburn, VA, USA) [30]. Prior to imaging, cells were seeded on a cover glass in a 35 mm petri dish at an estimated density of 2 × 10^5^ cells/dish and incubated for 24 h before treatment with PBS and 5% PEGylated 1:1, 1:2 and 1:3 DC-chol/DOPE lipoplexes. After a 72 h treatment period, cells were fixed with 4% paraformaldehyde for SRS/TPEF imaging. Images were processed and analyzed using Fiji (ImageJ).

*Nuclear/cytoplasmic separation:* Transfection efficiencies of lipoplexes prepared by 1:1, 1:2 and 1:3 PEGylated DC-chol/DOPE liposomes were analyzed. SK-OV-3 cells were seeded overnight in T25 culture flasks with a density of 6 × 10^5^ cells per flask. After cells attached to the surface, eight groups (*n* = 3) including PBS control, free DNA, and PEGylated 1:1, 1:2 and 1:3 lipoplexes at 64 nM as well as at a 32 nM lipid concentration, were added to the flasks to incubate with the cells. The lipid to DNA ratio (1 μM lipid to 15 μg/mL DNA) was the same throughout the groups. After 72 h, the cells were collected, washed with cold PBS twice, and resuspended in hypotonic buffer in five prechilled microcentrifuge tubes. After incubation on ice for 30 min with gentle agitation, the cells were centrifuged at 9000 rcf for 10 min at 4 °C. The pellet (nuclei) and the supernatant (cytoplasm) were stored separately in tubes on ice and analyzed using Spectramax i3 (Molecular Devices, San Jose, CA, USA) for fluorescence readings.

*In vitro pDNA transfection*: For transfection efficiency analysis, SK-OV-3 cells were seeded overnight in 24-well plates with a density of 2 × 10^5^ cells per well. The lipoplex containing 0.5 μg pDNA was incubated with the cells for 8 h until the fresh culture medium was changed. We used 0.5 µg plasmid DNA, an amount of DNA used in previous studies, as well as the protocol for Lipofectamine^TM^ LTX [31,32]. The plasmid DNA stock contained 25 µg DNA in 250 µL water (0.1 µg/µL). When treating cells, we used 5 µL of our stock, which equaled 0.5 µg of DNA, incubated with liposomes, and then added the liposome–DNA complex to the cells. After 72 h, the cells were trypsinized, washed and analyzed by flow cytometry (FCM). For the serum-free transfection, the medium was replaced with serum-free culture medium before transfection and 8 h after transfection, the medium was changed with culture medium containing serum. Transfection using Lipofectamine^TM^ LTX was performed according to the manufacture’s standard protocols and was used as a positive control. The pDNA transfection efficiency was determined with FCM (Becton-Dickinson, San Jose, CA, USA), using 488 nm excitation to detect the green light of GFP of transfected cells. The transfection efficiency was determined as the percentage of the transfected cells against all cells counted.

*Statistical analysis:* All *p* values were calculated using the Microsoft Excel *t*-test (two-sample assuming unequal variances) function (Redmond, WA, USA).

## 3. Results and Discussion

*Size and zeta potential of the blank DC-chol/DOPE liposomes and DNA-loaded liposomes (lipoplexes):* Before complex formation with DNA, all prepared DC-chol/DOPE liposomes had an average particle size of 133.9 ± 3.3 nm (Table 1). The average size of the lipoplexes was much larger than that of the liposomes from which they were produced (Table 1). At every DC-chol/DOPE molar ratio, the average size of PEGylated lipoplexes was significantly smaller (*p* < 0.05) than nonPEGylated lipoplexes, indicating that PEGylation could decrease the size of the lipoplex [32,33]. This decrease in size of PEGylated lipoplexes is attributed to the packing effects of PEG chains, which results in vesicle structures [34,35,36]. Furthermore, PEGylation had an impact on the homogeneity of the lipoplex nanoparticles, as the polydispersity index (PDI) values of PEGylated lipoplexes were significantly (*p* < 0.05) smaller than their nonPEGylated counterparts. The size reduction and improved homogeneity from PEGylation could be beneficial to efficient and stable transfections, as homogeneous small-size complexes are better internalized and processed by cells [37,38]. Zeta potential is another important parameter of cationic liposomes. As shown in Table 1, incorporation of PEG into cationic liposomes results in a reduction in the absolute value of the zeta potential, suggesting that PEGylation reduces the surface charge density. Furthermore, empty DC-chol/DOPE liposomes showed a positive surface charge of around 30 mV, whereas the surface charge of lipoplexes decreased to about 20 mV due to the electrostatic interactions between cationic lipids and anionic DNA. All the blank PEGylated and nonPEGylated liposomes were tested using a Zetasizer (Malvern Panalytical, Malvern, UK) after being stored at 4 °C for 7 days. All the formulations showed less than 20% change in size and PDI, indicating good colloidal stability.

**Figure 1:**
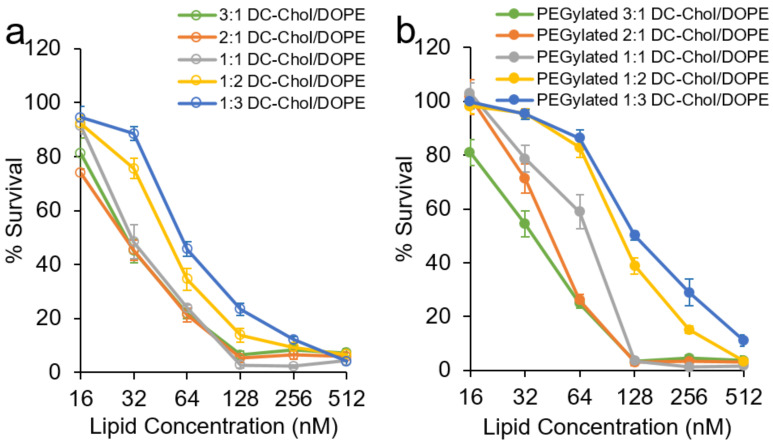

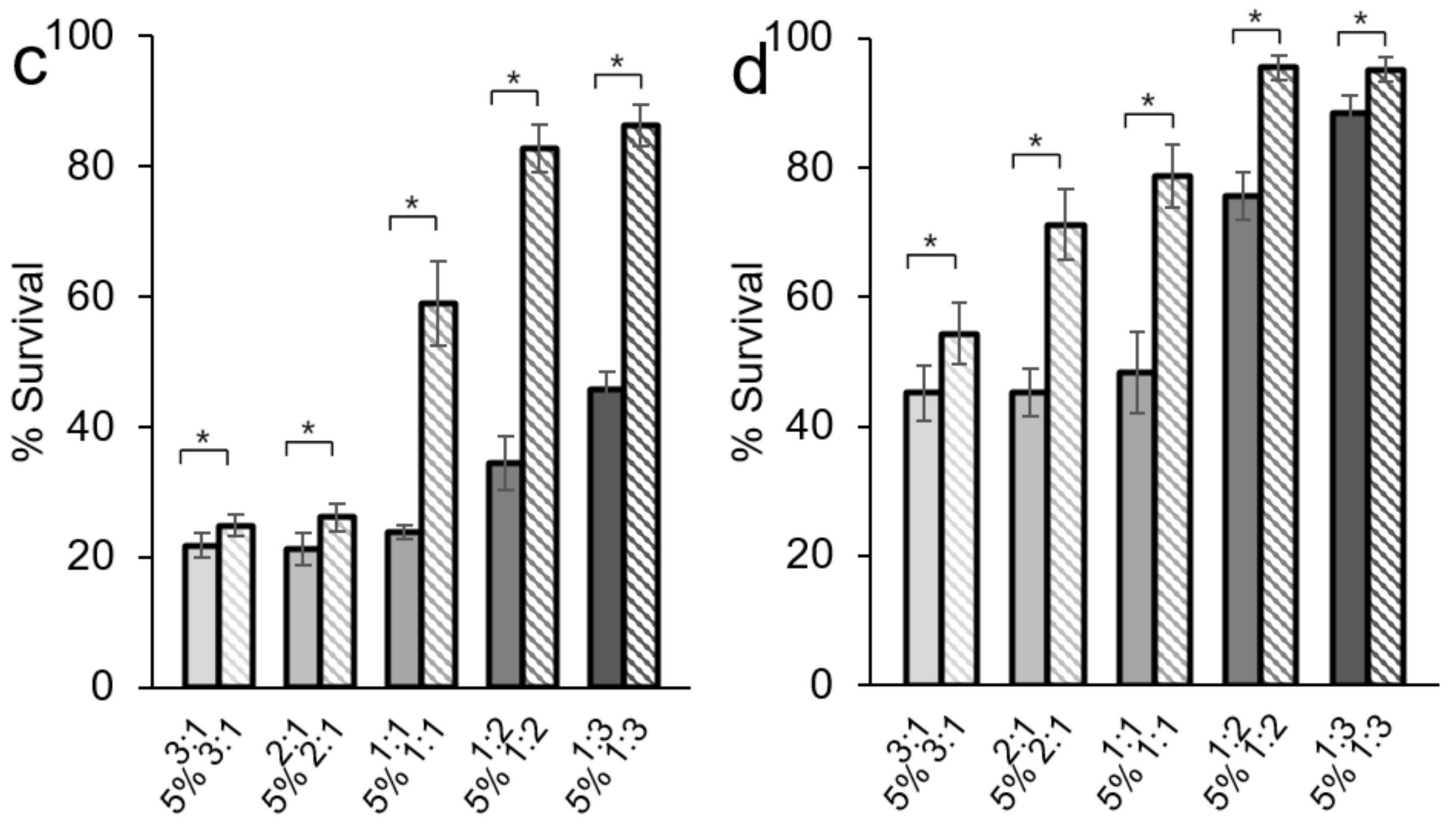
Percentage survival of cells treated with: (**a**) nonpolyethylene glycol (PEG)ylated 3:1, 2:1, 1:1, 1:2 and 1:3 Dc-chol/DOPE liposomes; (**b**) PEGylated 3:1, 2:1, 1:1, 1:2 and 1:3 Dc-chol/DOPE liposomes; (**c**) 5% PEGylated or nonPEGylated 3:1, 2:1, 1:1, 1:2 and 1:3 Dc-chol/DOPE liposomes at 64 nM and (**d**) 5% PEGylated or nonPEGylated 3:1, 2:1, 1:1, 1:2 and 1:3 Dc-chol/DOPE liposomes at lipid concentrations of 32 nM for 72 h. Data represented as mean ± SD (*n* = 4). Statistically significant (*p* < 0.05) differences are shown as *.

*Cytotoxicity of DC-chol/DOPE liposomes:* A recent study reported that many commonly used commercial transfection reagents, including Fugene and Lipofectamine 2000, exhibited high toxicities [39]. In this study, the cytotoxicities of the blank nonPEGylated and PEGylated 3:1, 2:1, 1:1, 1:2 and 1:3 DC-chol/DOPE liposomes were tested. SK-OV-3 cells were treated with ten different formulations at a series of lipid concentrations (16, 32, 64, 128, 256 and 512 nM) for 72 h, and the viability evaluated in comparison to cells treated with PBS (pH = 7.4). At lipid concentrations of 128, 256 and 512 nM, all liposomal formulations were quite toxic (Figure 1a,b). When the lipid concentration was decreased to 64 nM (or below), cell viability improved. In all cases, the toxicities of PEGylated formulations toward SKOV-3 cells were significantly (*p* < 0.05) lower that of their unPEGylated counterparts (Figure 1c,d). Our finding that PEGylation reduces the toxic effects of liposomes is consistent with previous studies [31,40]. This result can be explained by the shielding effect of PEGylation on the charge of cationic groups [41]. It is also worth noting that the toxicity of the cells treated with PEGylated as well as nonPEGylated liposomes reduced with a decrease in the cationic lipid DC-chol in the formulation. As the molar ratio of DC-chol/DOPE decreased, the cell viability increased from 22% to 46% and 25% to 86% for nonPEGylated and PEGylated liposomes, respectively. Cytotoxicity from DC-chol has been confirmed in previous reports [24]. Considering all this, PEGylated 1:1, 1:2 and 1:3 liposomes were selected for further experiments.

**Figure 2:**
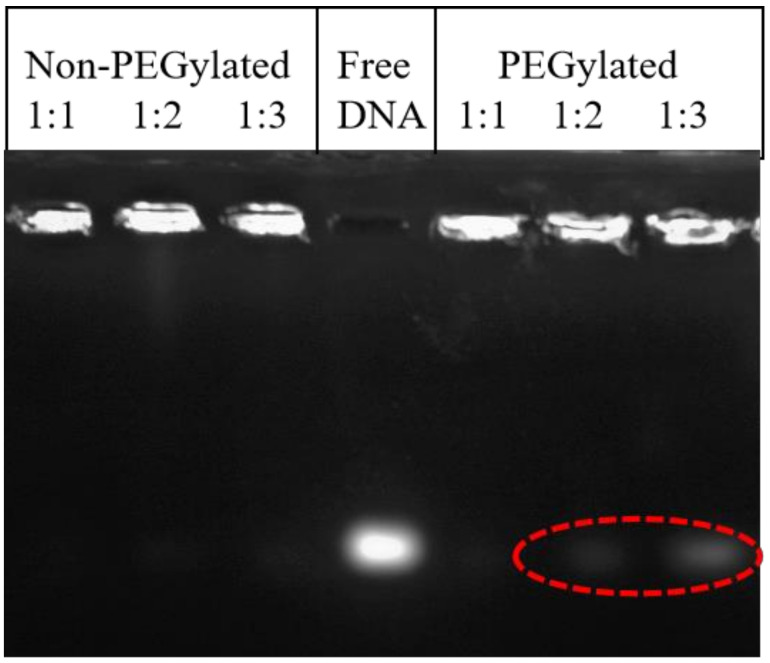
Agarose gel electrophoresis of nonPEGylated 1:1, 1:2 and 1:3 DC-chol/DOPE liposomes, free DNA, and PEGylated 1:1, 1:2 and 1:3 DC-chol/DOPE lipoplexes (from left to right).

*DNA binding affinity of DC-Chol/DOPE liposomes:* The gel retardation assay is the most commonly used method to examine the DNA binding affinity of cationic liposomes. The migration pattern of DNA in the PEGylated 1:1, 1:2 and 1:3 lipoplexes was compared to their nonPEGylated counterparts (Figure 2). No DNA migration was observed in nonPEGylated formulations and PEGylated 1:1 lipoplexes. However, a slight amount of DNA was detected for the PEGylated 1:2 and 1:3 lipoplexes. These results suggested that PEGylation in the 1:2 and 1:3 formulations can decrease the surface charge and show a negative effect with respect to DNA binding affinity, which is consistent with previous results [31,32,41].

**Figure 3:**
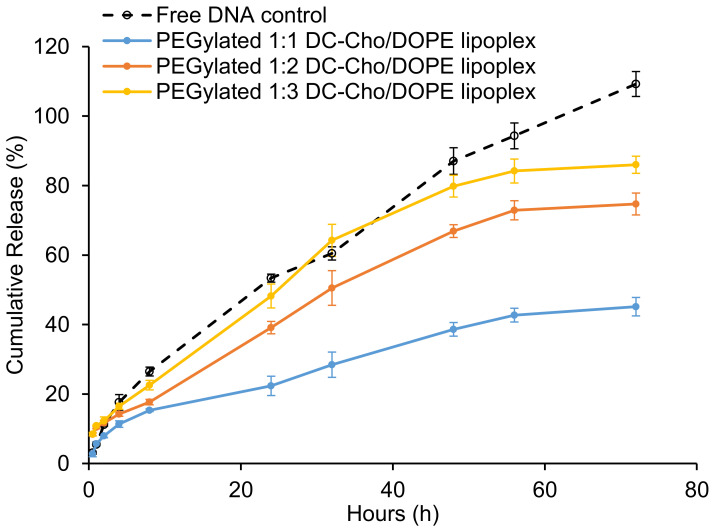
In vitro DNA release profile of free DNA (control). PEGylated 1:1, 1:2 and 1:3 DC-chol/DOPE lipoplexes over a 72 h period. Data represented as mean ± SD (*n* = 3).

*Release of DNA from the lipoplex:* It is necessary for the DNA to dissociate from lipids in order to have any efficacy, since its effects on expression cannot proceed when DNA and lipids are complexed together [42]. The in vitro release of DNA from the lipoplexes was studied over a 72 h period to examine the release efficiency in PBS (pH = 7.4) at 37 ± 0.5 °C. As shown in Figure 3, DNA was able to dissociate from three lipoplex preparations, and the amount of DNA released increased with the increase in DOPE in the formulation. The samples that have the fastest release profiles are also those that show unincorporated DNA in the gel. These two studies suggest that as DOPE increases, the binding between liposomes and DNA weakens, due to decreased electrostatic interactions. We performed release studies in PBS to determine if DNA could be released from the lipoplex. After demonstrating that it could, we used culture medium in all other studies.

**Figure 4:**
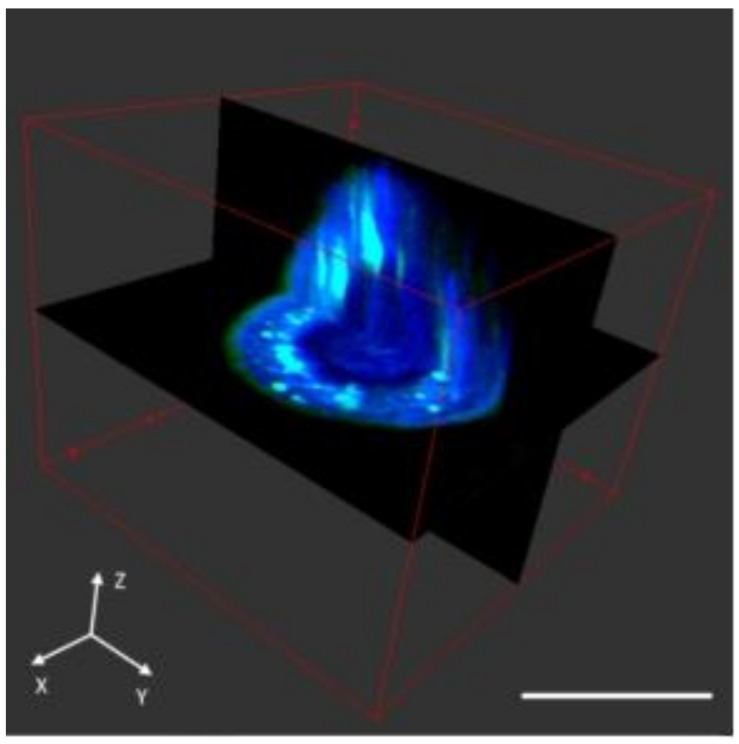
Three-dimensional volume reconstruction of a SKOV3 cell with z-stack Stimulated Raman Scattering (SRS) images taken with a depth interval of 0.2 μm. The green and blue pseudo colors represent lipids (imaged at 2854 cm^−1^) and proteins (imaged at 2930 cm^−1^), respectively. Scale bar: 20 μm. The 3D display was rendered using ImageJ.

*Visualization of the transfection efficiency of lipoplexes by SRS and TPEF microscopy:* The ability of the PEGylated 1:1, 1:2 and 1:3 lipoplexes to deliver DNA into the cells was studied using integrated Stimulated Raman Scattering (SRS) and Two-Photon Excitation Fluorescence (TPEF) microscopy. Both SRS and TPEF are multiphoton processes and their signals are only generated within the focal volume with the highest laser intensity. Therefore, the two imaging modalities show inherent optical sectioning abilities. The fine depth resolution of SRS and TPEF ensures that only an ultrathin layer (~1 μm) within the specimen will be imaged during a depth scanning event with nearly no signal generation out of the focal plane [43,44]. To validate the depth resolution of our imaging system, we imaged a single SK-OV-3 cell and acquired layer by layer stack images with depth intervals of 0.2 μm. As shown in Figure 4, we found that the spatial resolution was sufficient to distinguish the border of the cell nucleus in three dimensions with two-color SRS contrasts. In this regard, we can conclude that the slice images across the near-center of the cell nuclei should have no projection signals from outside the cell nuclei and, therefore, the slice images can be used to determine whether the cellular compartments or lipoplex are inside or outside of the cell nuclei.

**Figure 5:**
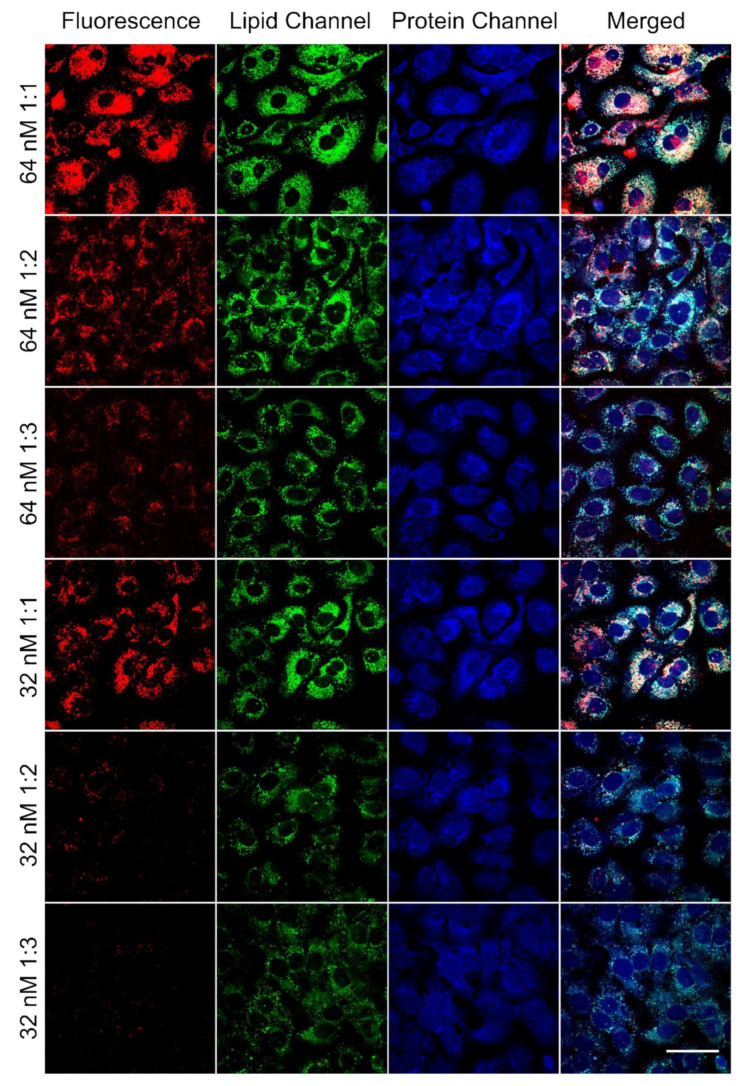
SRS and fluorescence images of SK-OV-3 cells treated with PEGylated 1:1, 1:2 and 1:3 DC-chol/DOPE lipoplexes at lipid concentrations of 64 and 32 nM. Lipid to DNA ratio (1 µM lipid to 15 µg/mL DNA) remained the same in 64 and 32 nM lipoplex groups. The images show overlap of the protein (pseudo blue), lipid (pseudo green), and FAM fluorescence (pseudo red) distribution in the cells. Scale bar: 50 µm.

Free of the interference of multiple labeling and excessive biological signals, the advantages of label-free SRS in identifying biomolecule distribution make it a useful tool to investigate the function of liposomes as a gene or drug delivery vehicle [45]. As shown in Figure 5, SRS images show the lipid and protein distribution and thus clearly outline the cytoplasm and nuclei of SK-OV-3 cells. It is exhibited in the FAM fluorescence (DNA) channel that the fluorescence intensity reduced with the decrease in the molar ratio of DC-chol/DOPE, suggesting the PEGylated 1:1 liposomes possess better transfection efficiency compared to PEGylated 1:2 and 1:3 liposomes. Furthermore, the change in the fluorescence signal of the three formulations was observed as being consistent with the change in the lipid droplets deposition, meaning that the gene transfection might be affected by the cellular uptake of lipoplexes. Containing more DC-chol in their formulations, 1:1 lipoplexes showed greater DNA delivery abilities as well as lipid internalization. The inclusion of cholesterol in lipoplex formulations has been reported to enhance transfection by affecting lipoplex internalization and intracellular trafficking [17,19,46]. In addition, cholesterol provides superior stability to lipoplexes in serum, thereby reducing the inhibitory effects caused by serum proteins [17]. As a cholesterol derivative, DC-chol may play a similar role in the enhancement of lipoplex internalization and stability, and thus contribute to higher transfection efficiencies. However, an excess amount of cationic lipid is detrimental concerning lipid toxicity to the cells [37]. Despite its capacity for gene delivery, PEGylated 1:1 DC-chol/DOPE was toxic to SKOV-3 cells (about 60% viable at 64 nM lipid concentration) and led to the formation of two nuclei in one cell, known as binucleation, which may cause a negative effect on subsequent mitosis [47]. Based on the previous results, we tested the toxicity of the liposomes at 32 nM (Figure 1d) and acquired acceptable cell viability of PEGylated 1:1 liposomes (approximately 80%). Then, SRS and TREF images at this concentration were taken and binucleation had almost disappeared (Figure 5).

**Figure 6:**
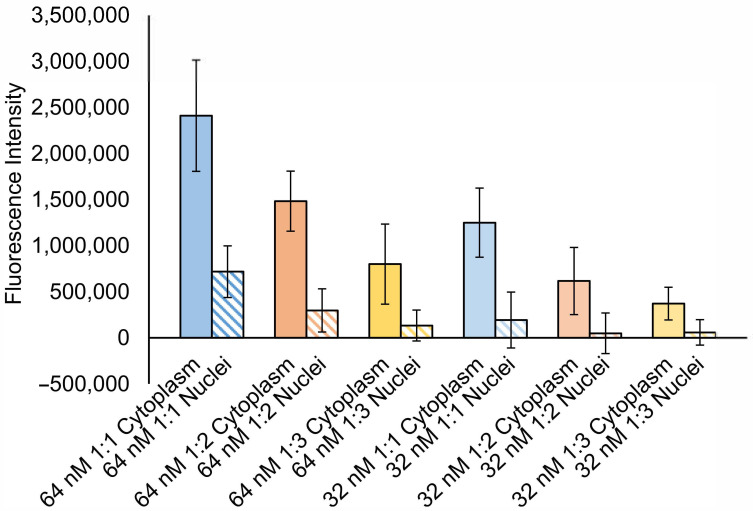
Nuclear and cytoplasmic fluorescence readings (background readings of Phosphate Buffer Saline (PBS) treated cells subtracted) of SK-OV-3 cells incubated with PEGylated 1:1, 1:2 and 1:3 DC-chol/DOPE lipoplexes at lipid concentrations of 64 and 32 nM. Data represented as mean ± SD (*n* = 3).

*Quantification of DNA delivery ability of lipoplexes by nuclear/cytoplasmic separation experiment:* A quantitative experiment was next performed to show the amount of DNA delivered into cells, and more precisely, to nuclei, by PEGylated 1:1, 1:2 and 1:3 lipoplexes at 64 and 32 nM. We harvested SK-OV-3 cells after treatment with the six groups for 72 h and isolated the cytoplasmic and nuclear components to read their fluorescence intensities using fluorescence spectrometry. As shown in Figure 6, similar to what was observed in the microscopy images, the fluorescence signal in the cytoplasm and nuclei decreased as the molar ratio decreased from 1:1, 1:2 to 1:3 for both lipid concentrations. The fluorescence intensities of both nuclei and cytoplasm treated with 1:1 lipoplexes were significantly higher than that of 1:2 lipoplexes. In terms of the comparison between 1:2 at 64 nM and 1:1 at 32 nM, the former showed higher FAM intensity from both cytoplasm and nuclei, however, the differences were not significant.

**Figure 7:**
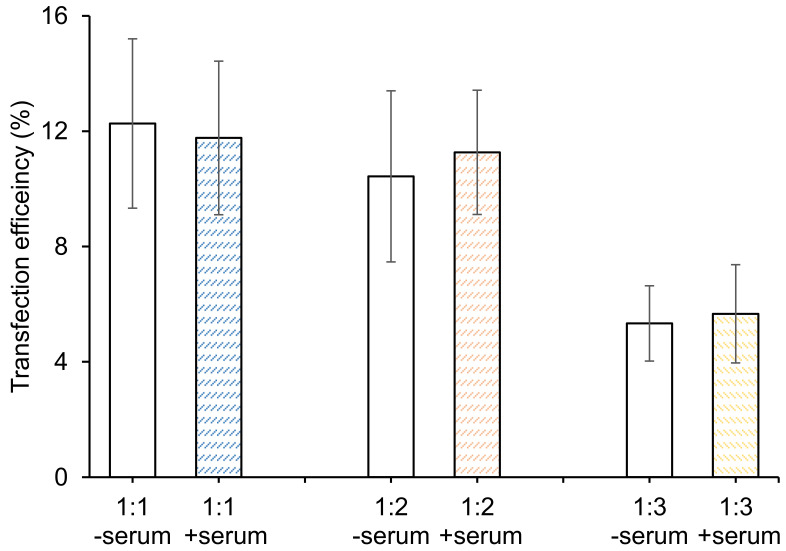
In vitro pDNA transfection assays. SK-OV-3 cells were seeded overnight in 24-well plates at a density of 2 × 10^5^ cells per well. PEGylated 1:1, 1:2 and 1:3 DC-chol/DOPE liposomes (all formulations had the same lipid ratio of 32 nM) were complexed with pDNA (a fixed amount of 0.5 µg). Then, the lipoplexes were incubated with SK-OV-3 cells for 8 h until fresh culture medium was changed. Seventy-two hours later, the cells were trypsinized, washed, and analyzed by flow cytometry. For serum-free transfections, the medium was replaced with serum-free culture medium and, 8 h after transfection, the medium was changed with culture medium containing serum. Data are presented as mean ± SD (*n* = 3).

*In vitro pDNA transfection:* We chose an oligonucleotide for the aforementioned studies because we are developing a delivery system for this type of construct; however, we do not know the functional outcome of this oligonucleotide in SK-OV-3 cells. Thus, to determine transfection efficiency, we performed a transfection study with plasmid DNA, which has a known functional outcome. Furthermore, we studied this in serum and serum-free environments. PEGylated 1:1, 1:2 and 1:3 DC-chol/DOPE liposomes were selected to incubate with a GFP-expressing plasmid to investigate their transfection efficiency and the effect of serum on transfection. As shown in Figure 7, PEGylated 1:1 and 1:2 exhibited similar transfection efficiencies while 1:3 lipoplexes had much lower efficiencies. No significant difference was found in serum-containing or serum-free pDNA transfection, suggesting that serum has little to no effect on PEGylated DC-chol/DOPE liposome-mediated pDNA transfection, which is consistent with previous studies [31,32]. The pDNA transfection experiments were performed at a 32 nM lipid concentration, because the cells used were >80% viable when exposed to all three of these formulations at this concentration. Lipofectamine^TM^ LTX was used as a positive control in this study, and a 27% transfection efficiency was observed. It has been reported that PEGylation may reduce transfection efficiency in vitro, which may be the reason for reduced transfection, compared to the positive control [31,32]. However, other variables cannot be ruled out.

## 4. Conclusions

The effects of inclusion of PEG and using different molar ratios of DC-chol/DOPE on size, polydispersity index, zeta potential, cytotoxicity and gene transfection efficiency of DC-chol/DOPE liposomes were reported here. In summary, PEGylation reduces the cytotoxicity of DC-chol/DOPE liposomes, and also leads to a smaller average size and narrower size distribution when these liposomes are complexed with DNA. Furthermore, careful consideration should be taken regarding the percentage of DC-chol included in the formulation to balance biocompatibility and transfection efficiency. It is also worth noting that both oligonucleotide and plasmid DNAs can be delivered into the nucleus by these liposomes, demonstrating that this system has potential for use with both single and double-stranded DNA. At this stage, we have explored the ability of these liposomes to work in vitro. The in vivo application of these formulations remains to be explored but is warranted based on these data.

## Figures and Tables

**Table 1:** Particle size distribution, polydispersity index (PDI) and zeta potential of 3-[N-(N’,N’-dimethylaminoethane)-carbamoyl] cholesterol (DC-chol)/dioleoylphosphatidylethanolamine (DOPE) liposomes and lipoplexes.

DC-chol/DOPE Ratios	DNA Loaded (µg/mL)	PEGylation	Size (nm)	PDI	Zeta Potential (mV)
3:1	0	0	134.4 ± 1.9	0.08 ± 0.01	38.3 ± 0.1
2:1	0	0	134.2 ± 3.1	0.07 ± 0.01	38.7 ± 0.6
1:1	0	0	137.5 ± 2.7	0.08 ± 0.01	38.6 ± 1.0
1:2	0	0	132.4 ± 6.7	0.08 ± 0.01	37.3 ± 1.1
1:3	0	0	136.3 ± 1.8	0.07 ± 0.01	35.5 ± 0.7
3:1	0	5%	126.4 ± 1.9	0.08 ± 0.01	28.2 ± 1.7
2:1	0	5%	132.6 ± 1.9	0.07 ± 0.02	27.5 ± 0.8
1:1	0	5%	137.3 ± 2.6	0.09 ± 0.02	27.5 ± 0.1
1:2	0	5%	135.4 ± 3.5	0.09 ± 0.01	30.6 ± 1.1
1:3	0	5%	132.4 ± 2.2	0.07 ± 0.01	28.6 ± 1.3
3:1	150	0	290.4 ± 7.2	0.14 ± 0.01	20.0 ± 0.6
2:1	150	0	293.6 ± 3.6	0.16 ± 0.02	20.0 ± 0.5
1:1	150	0	281.9 ± 5.6	0.16 ± 0.01	20.7 ± 1.1
1:2	150	0	292.1 ± 6.3	0.16 ± 0.03	20.3 ± 0.9
1:3	150	0	299.7 ± 5.1	0.15 ± 0.02	19.6 ± 0.6
3:1	150	5%	189.4 ± 4.0	0.13 ± 0.02	16.9 ± 0.8
2:1	150	5%	194.6 ± 4.8	0.10 ± 0.01	17.1 ± 0.7
1:1	150	5%	173.6 ± 6.9	0.10 ± 0.02	18.1 ± 1.1
1:2	150	5%	198.9 ± 6.1	0.10 ± 0.01	17.7 ± 0.2
1:3	150	5%	201.5 ± 4.5	0.10 ± 0.01	16.6 ± 0.5

Data represented as mean ± SD (*n* = 3).

## Data Availability

Data supporting the results can be obtained by contacting the authors.

## Abbreviations

DC-chol	3-[N-(N’,N’-dimethylaminoethane)-carbamoyl] cholesterol
DOPE	Dioleoylphosphatidylethanolamine
PEG	Polyethylene glycol
Lipoplex	Liposome–DNA complex
PBS	Phosphate Buffer Saline
PDI	Polydispersity Index
